# National health insurance accreditation pattern among private healthcare providers in Ghana

**DOI:** 10.1186/s13690-017-0205-9

**Published:** 2017-08-28

**Authors:** Abena Agyeiwaa Lamptey, Eric Nsiah-Boateng, Samuel Agyei Agyemang, Moses Aikins

**Affiliations:** 1 Dental Department, Pentecost Hospital, Madina, Accra, Ghana; 2 School of Public Health, College of Health Sciences, University of Ghana, Accra, Ghana

**Keywords:** Accreditation, Private healthcare providers, National health insurance scheme, Ghana

## Abstract

**Background:**

Healthcare providers’ accreditation is one of the standard means of assuring quality services. This paper examines the pattern of National Health Insurance Scheme accreditation results among private healthcare providers in Ghana.

**Methods:**

A cross-sectional quantitative analysis of administrative data from seven National Health Insurance Scheme healthcare provider accreditation surveys over the 2009–2012 period. Data on private healthcare providers that applied for formal accreditation between the study period were retrieved from the NHIS accreditation database using a checklist. Proportions were used to examine pattern of private healthcare provider accreditation results by region, type of care provider, and grade.

**Results:**

Overall, 1600 healthcare providers applied for accreditation over the study years, of which 1252 (78%) passed and were accredited. Majority of healthcare providers that passed the healthcare facility assessment were in Ashanti, Greater Accra, and Western regions, and were significantly higher than those in the other regions. Among the healthcare providers that passed the assessment, pharmacies (22%) and clinics (18%) constituted the largest groups, and were significantly higher than the other types of healthcare providers. Similarly, among those that passed, majority (62%) obtained grade C and D, representing a score of 50–59% and 60–69%, respectively, and were significantly higher than those that obtained the top three grades of A+ (90–100%), A (80–89%) and B (70–79%).

**Conclusions:**

Majority of healthcare providers accredited to provide services to the insured are concentrated in three regions of the country, and are mainly pharmacies and clinics. Moreover, substantial proportion of the healthcare providers obtain average scores of the healthcare facility assessment, an indication that these care providers fall below the National Health Insurance Scheme applicable-predetermined standards.

**Electronic supplementary material:**

The online version of this article (doi:10.1186/s13690-017-0205-9) contains supplementary material, which is available to authorized users.

## Background

Over the last decade, there has been an increased interest in development of accreditation programmes or tools for assessing healthcare providers and ensuring quality of care delivery in the health sector. This is being pioneered by international bodies involved in quality of care assessments to address quality of care challenges associated with increasing population and advancement in healthcare [1–3]. Other national governments in Africa have also initiated accreditation programmes to assess and monitor the quality of care delivery in their health systems in response to long waiting times, high cost, favouritism, disrespectful behaviour on the part of some healthcare providers, misuse and pilferage of medicines, and irregular availability of medicines, among others [4–6].

In Ghana, healthcare facility accreditation is a legal requirement for all care providers and the National Health Insurance Authority (NHIA) in collaboration with the Health Facility Regulatory Agency (HEFRA) undertakes this exercise [7–9]. HEFRA is mandated to register, licence, and monitor all healthcare facilities in the country. NHIA, on the other hand, credentials healthcare providers who have obtained accreditation from HEFRA and wish to provide services to the National Health Insurance Scheme (NHIS) members. The credentialing exercise is undertaken using applicable predetermined standard quality assessment tools as stipulated by the National Health Insurance Act, Act 852 and Legislative Instrument, LI 1809 [10, 11]. According to the NHIA, a total of 3434 healthcare providers have been accredited to provide services to the insured since July 2012 [12].

Since implementation of the NHIS policy in 2004, considerable achievements have been made in the area of providing financial access to healthcare for majority of Ghanaians [13–16]; however, geographical access and quality of care issues remain a challenge to both the insured and the uninsured. Review of the literature shows that there is no study on the NHIS that looked at the accreditation process and pattern of accreditation of healthcare providers. A study on NHIS accredited private and public primary health facilities was limited to efficiency of these healthcare providers [17]. Another study on the prospects and challenges of the NHIS briefly mentioned types and number of healthcare providers accredited to provide services and how they are reimbursed [13]. Other health quality related studies focused extensively on clients’ perception of quality of delivery under the scheme [15, 18], leaving very little information on the performance of private healthcare providers in the NHIS accreditation process. For example, information such as the trend of results as well as factors associated with success and failure in the accreditation process is not available. Therefore, this study examines the pattern of NHIS accreditation results among private healthcare providers. The significance is to advance the understanding and the necessity for accreditation as a standard regulatory practice to promote high quality of care among all NHIA service providers. Overview of the NHIS healthcare provider accreditation is provided in Additional file 1: Appendix 1.

## Methods

The first part of this section describes the process of the NHIA healthcare provider accreditation and performance score. The later part focuses on design of the study, the study population, and data collection techniques used.

### Healthcare provider accreditation and performance score process

The healthcare provider accreditation process begins with an application from interested healthcare providers (see Additional file 1: Appendix 2). A completed application form is submitted along with relevant documents and applicable fees. Applications are vetted to ensure that forms are appropriately filled, after which receipt and accreditation manual are issued. The manual issued to care providers are used to make necessary preparation for inspection. According to the unpublished accreditation manual, inspection (or direct observation) and scoring of facilities are conducted based on 12 assessment modules; (i) range of service; (ii) staffing; (iii) environment and infrastructure; (iv) basic equipment; (v) organisation and management; (vi) safety and quality management; (vii) out-patient care; (viii) in-patient care; (ix) maternity care; (x) specialised care; (xi) diagnostic services; and (xii) pharmaceutical services. Each module is divided into sub-units and all sub-units have a set of standards which depend on the type of facility (hospital/clinic, pharmacy/chemical shop, maternity home) and the level of service provision (primary, secondary, tertiary). An example of the standards tool for assessing “range of services” and “staffing” modules of clinics is shown in Additional file 1: Appendix 3. The category to which the standard belongs is indicated with the letter I (Input standard), P (Process standard), H (Human capacity), O (Output/Outcome standard) or S (Services) under the column labelled “category”. The criteria for assessing the standards and the methods of assessment are also indicated for each standard. In order to meet the requirement of a standard and be accredited, a healthcare provider should satisfy the criteria listed under the subheading “Definition”. The method of assessment is also indicated under the sub-heading “Methods”.

Scoring of each of standard is done according to the following criteria: Score 3 if all criteria are met; Score 2 if half or more are met but not all (≥½); Score 1 if less than half are met (but not zero); Score 0 if no criterion is met; and Score “N/A” if the standard is not applicable [8]. On average, a team of four observers with varied professional backgrounds in medical and health disciplines assesses one healthcare provider; however, the assessment is not blinded. Each unit/module score is obtained by averaging all the sub-unit scores. The 12 unit/module scores are then added up and the cumulative score determines the facility score on which the outcome is based. All the modules have the same weight for the scores. A healthcare provider is accredited if the facility score is 50% and above. Provisional accreditation is given when facility score is less than 50% but scores 50% and above in core areas put together. Accreditation is denied when a facility scores below 50% overall and/or in core areas put together. Facilities which are already accredited to provide service at a lower level may reapply for accreditation for a higher level. These facilities are assessed on modules based on the desired high level and when successful, an upgraded accreditation is given [8]. The interpretation of the assessment score is shown in Additional file 1: Appendix 4.

### Study design

This was a cross sectional quantitative study using administrative data from seven NHIS accreditation surveys covering the period, July 2009 to July 2012.

### Study population

All private healthcare providers that applied for NHIS accreditation between July 2009 and July 2012 were used for the study. They included pharmacies, chemical shops, maternity homes, laboratories, scan centres, clinics, primary hospitals and secondary hospitals. Other healthcare provider information were location (or address of healthcare facility), ownership, level of care, accreditation scores and accreditation status.

### Data collection and management

The NHIA accreditation and performance score data were extracted from the NHIA accreditation database using data extraction checklist. The completeness of the data was assessed and healthcare providers whose data were incomplete were contacted through telephone or by visit to obtain missing information.

### Data analysis

A descriptive analysis was employed to examine pattern of success (or pass) and failure among private healthcare providers (PHCPs), pattern of reaccreditation among PHCPs with provisional accreditation, and pattern of upgrade among PHCPs. The analysis was conducted by region and by type of healthcare provider for all the seven batches of accreditation surveys using Microsoft Excel (2010 version). Successful accreditation per batch was estimated as proportion of accredited (passed) healthcare provider per accreditation batch; failure per batch was calculated as proportion of failed healthcare provider per accreditation batch; successful reaccreditation per batch was determined as proportion of accredited healthcare provider with provisional accreditation per batch; and successful upgrade per batch was estimated as proportion of successful upgrade per upgrade application. Confidence intervals were also estimated for proportion of healthcare providers that passed the accreditation assessments, using Stata immediate command “cii N X, level (95)”; where cii is immediate confidence interval, N is sample size or number of observations, and X is number of successes [19].

## Results

### Distribution of the application data

**Fig. 1:**
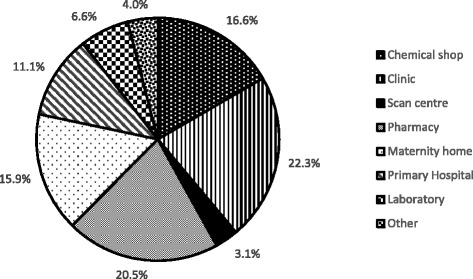
Distribution of applications by healthcare facility type, 2009–2012

A total of 1600 applications were received for accreditation between July 2009 and July 2012, of which clinics constituted 356 (22.3%), pharmacies, 328 (20.5%) and chemical shops, 266 (16.6) (Fig. 1). About 16% (254) were from Maternity homes while the least applications were from Scan or Diagnostic centres 3.1% (50).

### Distribution of accreditation application and performance score by region

Overall, one hundred and ten (110) applications were submitted over the study period for reaccreditation (renewal of accreditation), reapplication (subsequent applicants with provisional accreditation or those who failed previous assessments), upgrade to next level of care, and downgrade (Table 1). Out of this number, Ashanti region submitted the highest of 41 applications, of which 20 (32%) were reapplications, 16 (39%) reaccreditation, and 5 (12%) upgrades. Twenty-nine applications came from the Brong-Ahafo Region, of which 20 (69%) were reapplications, 7 (24%) reaccreditations and 2 (7%) upgrades. The Upper East Region had only two reapplications, (1 reapplication and 1 reaccreditation). There were no reapplications from the Upper West Region. All applications for upgrade passed except for one failed application from the Brong-Ahafo region. Only two healthcare providers in the Eastern region were downgraded.

**Table 1:** Application and performance score by region, 2009–2012

	Upgrade		Downgrade		Reaccreditation		Reapplication		
Region	Pass (%)	Fail (%)	Pass (%)	Fail (%)	Pass (%)	Fail (%)	Pass (%)	Fail (%)	Total
Ashanti	5(12.2)	0(0.0)	0(0.0)	0(0.0)	13(32.0)	3(7.3)	16(39.0)	4(9.8)	41
Brong-Ahafo	1(3.5)	1(3.5)	0(0.0)	0(0.0)	3(10.4)	4(13.8)	20(67.0)	0(0.0)	29
Central	1(16.7)	0(0.0)	0(0.0)	0(0.0)	2(33.3)	0(0.0)	3(50.0)	0(0.0)	6
Eastern	1(16.7)	0(0.0)	2(33.3)	0(0.0)	0(0.0)	0(0.0)	3(50.0)	0(0.0)	6
Greater Accra	1(33.3)	0(0.0)	2(66.7)	0(0.0)	0(0.0)	0(0.0)	0(0.0)	0(0.0)	3
Northern	0(0.0)	0(0.0)	0(0.0)	0(0.0)	0(0.0)	1(25.0)	3(75.0)	0(0.0)	4
Upper East	0(0.0)	0(0.0)	0(0.0)	0(0.0)	0(0.0)	1(50.0)	1(50.0)	0(0.0)	2
Upper West	0(0.0)	0(0.0)	0(0.0)	0(0.0)	0(0.0)	0(0.0)	0(0.0)	0(0.0)	0
Volta	0(0.0)	0(0.0)	0(0.0)	0(0.0)	1(7.7)	0(0.0)	6(46.2)	6(46.2)	13
Western	4(66.7)	0(0.0)	0(0.0)	0(0.0)	1(16.7)	0(0.0)	1(16.7)	0(0.0)	6

### Healthcare provider performance score by region

**Fig. 2:**
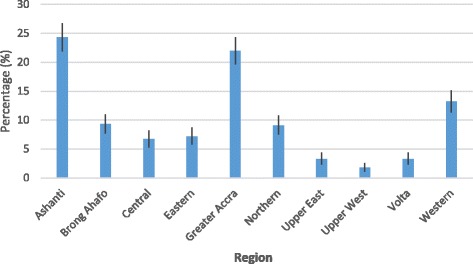
Proportion of healthcare providers with accreditation (95% CI) by region, 2009–2012

Out of the 1600 accreditation applications assessed, 1252 (78%) applications passed while 348 (22%) failed. Accreditation performance score by region showed that Ashanti region had the highest number of healthcare providers that passed, 304 (24%; 95%CI: 21.9%–26.7%), followed by Greater Accra region, 275 (22%; 95%CI: 19.6%–24.3%) and Western region, 165 (13%; 95%CI: 11.3%–15.1%) (Fig. 2). The proportion of healthcare providers in Ashanti, Greater Accra, and Western regions that passed the healthcare facility assessment were significantly higher than those in the other regions (CIs did not overlap either of the other regions). Likewise, there were significant differences in the proportion of healthcare providers that passed the healthcare facility assessment between Ashanti and Western, and Greater Accra and Western. However, there were no significant differences in the proportion of healthcare providers that passed the healthcare facility assessment in Ashanti and Greater Accra; Brong-Ahafo and Central; Brong-Ahafo and Eastern; Eastern and Central, Northern and Eastern, and Upper East and Upper West, as lower and upper bound limits overlapped each other, respectively.

### Healthcare provider performance score by type of facility

**Fig. 3:**
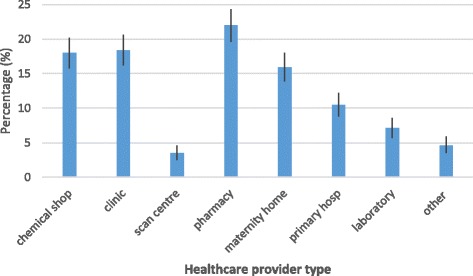
Proportion of healthcare providers with accreditation (95% CI) by type of facility, 2009–2012

The healthcare provider performance assessment by type showed that pharmacy recorded the highest proportion that passed the assessment, 275 (22%; 95%CI: 19.6%–24.3%), followed by clinic 230 (18%; 95%CI: 16.2%–20.6%), and chemical shop, 225 (18%; 95%CI: 16.2%–20.2%) (Fig. 3). The proportion of pharmacies, clinics and chemical shops that passed the healthcare facility assessment were significantly higher than that of scan centre, laboratory, primary hospital, maternity home and other facilities. However, there were no significant differences in the proportion that passed between pharmacy and clinic; pharmacy and chemical shop; clinic and chemical shop; clinic and maternity home; and chemical shop and maternity home, as lower and upper bound limits overlapped each other, respectively.

### Healthcare provider performance score by grade

**Fig. 4:**
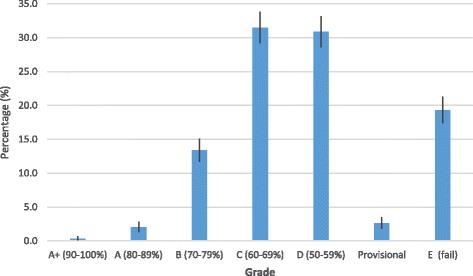
Proportion of healthcare providers with accreditation by grade, 2009–2012

Accreditation performance score by grade showed that 251 (16%) healthcare providers obtained the top three grades (A+, A, B) while 1349 (84%) obtained the lowest four grades (C, D, Provisional or fail) (Fig. 4). Majority of the healthcare providers, 504 (32%; 95%CI: 29.2%–33.8%) obtained grade C; 494 (31%; 95%CI: 28.6%–33.2%) obtained grade D; and 309 (19%; 95%CI: 17.4%–21.3%) obtained grade E (fail). The proportion of healthcare providers that obtained grade C and D were significantly higher than those that obtained A+, A, B, provisional and E. Likewise, the proportion of healthcare providers that obtained grade E (fail) were significantly higher than those that obtained grade A+, A, B, and provisional. However, healthcare providers that obtained grade C and D showed no significant difference because the lower limit of grade C overlapped with the upper limit of grade D.

## Discussion

This study sought to examine the pattern of NHIS accreditation results among private healthcare providers over the 2009–2012 period. The findings show that pharmacies and clinics constitute the largest groups of healthcare providers accredited to provide services to the insured. This result was expected due to the large number of these private healthcare providers across the country compared to the public healthcare providers, which are exempted from the accreditation process. The positive effect is that, majority of these accredited healthcare providers are found in the remote areas of the country where there are limited number of public/government healthcare providers. For instance, in some areas the only government healthcare provider that may be available is the Community-based Health Planning and Services (CHPS) compound. Therefore, the high proportion of pharmacies and clinics could help address the challenge of geographical access to healthcare services, as well as geographical equity in access to healthcare in deprived communities.

Assessment of applications by administrative region reveals significant differences in the proportion of healthcare providers that passed. In all, Ashanti region submitted the highest number of applications and also had the highest proportion of healthcare providers that passed the accreditation assessment. This was followed by the Greater Accra and Western regions. The proportion of healthcare providers in these three regions that passed the healthcare facility assessment were significantly higher than those from the other seven regions. Whilst proportion of healthcare providers in Ashanti and Greater Accra region that passed the healthcare facility assessment show no significant difference, there were significant difference between Ashanti and Western region. These results are expected due to the proportionally large number of private healthcare providers in these three regions in the country. The significance of these results is that it would facilitate easy access to healthcare provider services for the substantial number of the insured residing in these three regions. Similarly, the relatively large number of accredited healthcare providers in the Brong-Ahafo region could help address the challenge of geographical access to healthcare in the remote communities. However, the small number of healthcare providers in the three deprived regions of the country (Northern, Upper East, Upper West) that passed the healthcare facility assessment could pose a barrier to access to health services for the insured in these regions.

Results of the study also show significant differences in the proportion of healthcare providers that passed the healthcare facility assessment by type of provider. Majority of the pharmacies, clinics and chemical shops passed the assessment; however, there were no significant differences between them. On the contrary, proportion of pharmacies that passed the assessment were significant higher than that of the other types of healthcare providers (scan centres, maternity homes, primary hospitals, and laboratories). Interestingly, the clinics also recorded the highest number of failed applications, which means that most of them fall below the NHIS applicable pre-determined standards for accreditation.

Findings of the study also show that among the healthcare providers that passed the healthcare facility assessment and were accordingly accredited, about two-thirds had average score of 50% to 69% (grade C and D), and were significantly higher than those that obtained a score of 70% to 100% (grade A+, A and B). Only a limited number of healthcare providers (about one-sixth) obtained the top three scores of the accreditation assessment. The plausible reason is that majority of the healthcare providers’ facilities are below the pre-determined standards. This finding supports a study by Alhassan et al. [17], where less than one-third of NHIS accredited private primary healthcare facilities were found to be optimally efficient. Our finding implies that majority of the healthcare facilities are not well-resourced, and this could result in limited access to needed resources such as personnel and technology, as well as important services including laboratory and imaginary.

The large number of healthcare providers in the score bracket of 50–69% also raises the question of “is it the case of accreditation fraud, where healthcare providers borrow or rent equipment and other resources for the purpose of satisfying the inspection team to get the desired level of accreditation and return them after the inspection? Or is it the case where healthcare providers that do not meet the pre-established standards for accreditation tend to “buy” the inspecting team, as found in other study [6]? Or the NHIA facility inspection team applies collegial approach of accreditation to some of the healthcare providers especially those that do not meet the applicable requirements for accreditation? In the last instance, the team may favour the unqualified facilities by given average or weak performance score, as found in other studies [1, 5, 19, 20]. One key measure that NHIA uses to address the issue of healthcare providers borrowing or renting equipment and other resources in order to gain accreditation is post accreditation monitoring, involving a team of experts drawn from the health sector. However, the large number of care providers with the average score needs to be examined further to ensure that only providers that meet the applicable pre-determined standards are accredited to render services to the insured.

### Study limitations

There were gaps in the data obtained especially information on reapplication. Thus, deductions had to be made on which applications were for reaccreditation (or renewal) and which ones were reapplications from healthcare providers that did not meet requirements of previous healthcare facility assessment exercises, making it a potential source of error. Secondly, the study used a 5-year old data; hence, generalisation of the findings needs to take this into account.

## Conclusions

The study reveals significant differences in accreditation scores between regions and between healthcare providers. Healthcare providers in the Ashanti, Greater Accra and Western regions recorded significantly higher assessment scores than those in the other seven regions of the country. Accreditation by type of healthcare provider also shows that pharmacies, clinics, and chemical shops obtained significantly higher scores than the other types of healthcare providers; however, they are no significant differences in pass scores between them. The study also reveals that majority of the healthcare providers that apply for accreditation obtain average assessment scores, and are significantly higher than those that obtain the top three scores. This suggests a need for regulatory authorities to enforce standards to ensure provision of quality care and better health outcomes for the population. Further study would also be necessary to assess accredited healthcare providers’ level of adherence (or compliance) to treatment protocols governing their accreditation status.

## Additional file

Additional file 1: Appendix 1. Overview of NHIS healthcare provider accreditation system. **Appendix 2.** NHIS accreditation cycle. **Appendix 3.** Range of services and staffing standards for assessing clinics. **Appendix 4**. NHIS accreditation score interpretations. (DOCX 672 kb)

## Abbreviations

CI: Confidence interval
DMHIS: District mutual health insurance scheme
HEFRA: Health facility regulatory agency
MOU: Memorandum of understanding
NHIA: National Health Insurance Authority
NHIS: National Health Insurance Scheme
PHCP: Private Healthcare Services Provider

## References

[1] Shaw CD. Some issues in the design and redesign of external health care assessment and improvement systems. Toolkit Accreditation Programs. Melbourne: The International Society for Quality in Health Care; 2004.

[2] Shaw CD, Kutryba B, Braithwaite J, Bedlicki M, Warunek A (2010). Sustainable healthcare accreditation: messages from Europe in 2009. Int J Qual Heal Care.

[3] Montagu D. Accreditation and other external quality assessment systems for health care. DFID Heal Syst Resour Cent Work. 2003. http://hdrc.dfid.gov.uk/wp-content/uploads/2012/10/Accreditation-and-other-external-quality-assessments.pdf. Accessed 13 Aug 2016.

[4] Quality Assurance Project. The Zambia Accreditation Program Evaluation Accreditation Program. Oper Res Results. 2005. http://pdf.usaid.gov/pdf_docs/Pdacg129.pdf. Accessed 13 Aug 2016.

[5] Mate KS, Rooney AL, Supachutikul A, Gyani G (2014). Accreditation as a path to achieving universal quality health coverage. Glob Health.

[6] Ministry of Health. Accreditation of Providers for the National Health Insurance Fund of Tanzania. 1999.

[7] Republic of Ghana. National Health Insurance Regulations, 2004 ( Li 1809 ). Regulation, 1809 Accra, Ghana; 2004.

[8] Tweneboa NA. NHIS accreditation in Ghana. In Cape Town; 2011 http://www.cohsasa.co.za/sites/cohsasa.co.za/files/dr_nicholas-tweneboa.pdf. Accessed 15 Aug 2016.

[9] Nathaniel Otoo. HFRA: A structured approach from Ghana. In Mombasa; 2013 http://www.safe-care.org/uploads/4.%20Nathaniel%20Otoo%20-%20HFRA%20a%20structured%20approach%20fromGhana%20[Compatibility%20Mode].pdf. Accessed 15 Aug 2016.

[10] Republic of Ghana. National Health Insurance Act, 2012 (Act 852). Accra, Ghana: Parliament of Ghana; 2012.

[11] Republic of Ghana. National Health Insurance Act, 2003 (Act 650). Insurance Act, 650 Accra, Ghana; 2003.

[12] National Health Insurance Authority. Annual report. Accra; 2013. http://www.nhis.gov.gh/. Accessed 14 May 2016.

[13] Gobah FK, Zhang L (2011). The National Health Insurance Scheme in Ghana: prospects and challenges: a cross-sectional evidence. Glob J Health Sci.

[14] Nsiah-boateng E, Aikins M, Asenso-boadi F, Andoh-Adjei F-X (2016). Value and service quality assessment of the National Health Insurance Scheme in Ghana: evidence from Ashiedu Keteke District. Value Heal Reg Issues.

[15] Dalinjong PA, Laar AS (2012). The national health insurance scheme: perceptions and experiences of health care providers and clients in two districts of Ghana. Health Econ Rev.

[16] National Development Planning Commission. 2008 CITIZENS ’ ASSESSMENT OF THE NATIONAL HEALTH INSURANCE SCHEME: Towards a Sustainable Health Care Financing Arrangement that Protects the Poor. Accra; 2009.

[17] Alhassan RK, Amponsah EN, Akazili J, Spieker N, Arhinful DK, De WTFR (2015). Efficiency of private and public primary health facilities accredited by the National Health Insurance Authority in Ghana. Cost Eff Resour Alloc.

[18] Atinga R (2012). Healthcare quality under the National Health Insurance Scheme in Ghana: Perspectives from premium holders. Int J Qual Reliab Manag.

[19] Stata.com. Confidence intervals for means, proportions, and counts. http://www.stata.com/manuals13/rci.pdf. Accessed 10 June 2017.

[20] Shaw CD, Braithwaite J, Moldovan M, Nicklin W, Grgic I, Fortune T (2013). Profiling health-care accreditation organizations: an international survey. Int J Qual Heal care.

