# Supplementary material for: National health insurance accreditation pattern among private healthcare providers in Ghana
Source: Arch Public Health. 2017 Aug 28;75:36. doi: 10.1186/s13690-017-0205-9 (PMC5572156; doi:10.1186/s13690-017-0205-9)
Supplement: Additional file 1: Appendix 1. — Overview of NHIS healthcare provider accreditation system. Appendix 2. NHIS accreditation cycle. Appendix 3. Range of services and staffing standards for assessing clinics. Appendix 4. NHIS accreditation score interpretations. (DOCX 672 kb) [file 13690_2017_205_MOESM1_ESM.docx]

**Appendix 1: Overview of NHIS healthcare provider accreditation system**

In 2005, when the NHIS became fully operational, the then District Mutual Health Insurance Schemes (DMHISs) started engaging healthcare providers in their respective districts to join the scheme to provide services to the insured. As limited by guarantee companies, the DMHISs were autonomous; thus, had their own governing body (or the board) providing oversight responsibility of NHIS operations at the district level [11]. First, the DMHISs began signing contracts with public healthcare providers that were mandated to join the scheme in providing services to the insured. The private healthcare providers including the faith-based healthcare facilities and community pharmacies that were willing to provide services to the insured were also contracted through memorandum of understanding (MOU). The MOUs with healthcare providers were for one year period, after which they could renew their contract with the schemes. These MOUs with care providers were meant to kick start implementation of the scheme [8,9].

The Legislative Instrument (LI, 1809) governing the operations of the NHIS in the early years spelt out qualifications and specific accreditation requirements for healthcare providers willing to provide services to the scheme [7]. However, there were no formal accreditation tools to assess the capacity of these providers within the stipulated requirements. Besides, the DMHISs also lacked the professional capacity to undertake healthcare facility assessment using established quality assessment tools or guidelines. These limitations resulted in challenges to operations of the scheme nationwide. There were arbitrary negotiations for tariff increases between DMHISs and their care providers, leading to disparities across the country and high claims costs which threatened financial sustainability of the NHIS. There were also provision of perceived poor quality care to the insured. Recognizing these challenges, the NHIA took over accreditation of healthcare providers in the year 2007.

In the 2007-2008 period, providers were asked to apply to the NHIA for accreditation. However, healthcare providers already providing services to the scheme were allowed to continue to do so whilst their applications were being assessed. After initial assessment of the applications, providers that qualified were given a 6-month provisional accreditation. Names of successful applicants were also published in the Daily Graphic Newspaper and official letters sent to them, as well. Accredited healthcare providers were required to send their letters to the DMHIS office in their districts and sign contracts with them before rendering services to the insured.

Since 2009, healthcare providers willing to provide services to the NHIS members are required to apply for formal accreditation and pay the appropriate fee [8,9]. Successful applicants are given a 5-year period for the first time, and two years for subsequent renewals. The NHIA has developed accreditation tools for assessing healthcare providers’ capacity (human resource, infrastructure, equipment, etc) to provide services to the insured. Post-accreditation measures have also been put in place to ensure continued adherence to accreditation requirements and provision of quality care to the insured. Healthcare providers who flout the requirements and conditions of their accreditation face suspension or revocation of accreditation over a maximum period of six months.

**Appendix 2: NHIS accreditation cycle**

**Appendix 3: Range of services and staffing standards for assessing clinics**

**Appendix 4: NHIS accreditation score interpretations**

| **Grade** | **Total facility score** |
| --- | --- |
| A+ | 90 - 100% |
| A | 80 -89% |
| B | 70 - 79% |
| C | 60 - 69% |
| D | 50 - 59% |
| Provisional | Facility score< 50% but total in core areas 50% or above |
| E = fail | Facility score< 50% and /or total in core areas< 50% |
